# Suicide mortality in Bangladesh: a comparative analysis of the incidence of suicide in 2002 and 2015 from Bangladesh Health and Injury surveys

**DOI:** 10.1186/s12888-026-07819-2

**Published:** 2026-01-28

**Authors:** Labida Islam, Koustuv Dalal, Shaheera Rahman, Shagoofa Rakhshanda, Salim Mahmud Chowdhry, AKM Fazlur Rahman, Saidur Rahman Mashreky

**Affiliations:** 1 Orbis International, Dhaka, Bangladesh; 2 Division of Public Health Science, Department of Health Sciences, Mid Sweden University, Sundsvall, Sweden & University of Johannesburg, Johannesburg, South Africa; 3 Brac University, Dhaka, Bangladesh; 4 University of New South Wales, Sydney, Australia; 5 Centre for Injury Prevention and Research Bangladesh, Dhaka, Bangladesh; 6 North South University, Dhaka, Bangladesh

**Keywords:** Suicide, Mortality rate, Bangladesh, Changes over time

## Abstract

**Background:**

Suicide ranks as the 17th highest cause of death worldwide, making it an ongoing public health concern. To comprehend Bangladesh’s current mental health situation, it is essential to compare the country’s suicide death rates across time. The current study compares the changes in the suicide mortality rates in Bangladesh over 13 years from 2002 to 2015. This is a cross-sectional independent-sample design comparison using two distinct nationally representative datasets.

**Methods:**

In Bangladesh, two community-based nationally representative surveys, namely the Bangladesh Health and Injury Survey (BHIS), were conducted in 2003 and 2016. Both surveys adopted a similar approach. To get the intended sample, a multistage cluster sampling approach was applied in both surveys, while considering the probability-proportional-to-size technique. The population’s causes of morbidity and mortality data were collected using a three-year recall period and compared with suicide occurring in 2002 and 2015 using a pretested, semi-structured questionnaire. The cause of death was determined by the verbal autopsy procedure.

**Result:**

The suicide rates in 2002 were 6.2 per 100,000 population (95% CI: 4.7–8.1); whereas in 2015, it was 7.7 per 100,000 population (95% CI: 5.1–11.6). A major shift in suicide trends among the age group was observed between 2002 and 2015. Results showed that those aged 60 years and above had the highest suicide rates in 2002 (rate: 13 per 100,000; 95% CI: 2.6–23.4) and in 2015, adolescents had the highest suicide rates (rate: 22.9 per 100,000; 95% CI: 13.6–38.7). Moreover, in 2015, females had a higher suicide rate compared to males, but in 2002, both sexes showed similar death rates. In both years, the death tolls were higher in the rural areas than in the urban areas.

**Conclusion:**

The suicide rates have been increasing over the past decade, which is alarming for the nation. Extensive research is needed now to explore the factors affecting the increasing suicide rates among adolescents and females.

## Introduction

Suicide is a major public health concern globally as it is the 17^th^ leading cause of death across the lifespan and is ranked as the fourth leading cause of death among people aged 15–29 years [1]. According to the estimation made by the World Health Organization (WHO), suicides account for approximately 720,000 deaths annually, which makes suicide one of the leading causes of death [2]. Suicide not only causes a loss of life, but it also has more dreadful impacts. It harms the economy and community as well as the family of the bereft [3, 4].

In Asian countries, the condition is comparatively more dire, as 11 low-middle-income countries (LMICs) in Southeast Asia account for 26% of the global population but bear a striking 39% of global suicides [5]. The suicide rate in Southeast Asia is approximately 17.7 per 100,000 individuals, which is the global highest [6]. As a result, it is of utmost importance to understand the sociocultural, economic, and environmental factors that contribute to such remarkably high rates of suicide in this region. Furthermore, LMICs account for a significant portion of the world’s population, and it has been observed that LMICs contribute to approximately 80% of global suicides [7]. However, intervention and prevention strategies for suicide are not very developed in such countries, which need to be taken into consideration [8].

Accurate data must be collected and analyzed regarding the rate of suicide worldwide; however, the quality of data on mortality caused by suicide varies globally, as many countries do not have proper reporting systems [9]. This lack of precise data hinders the development of proper intervention and prevention strategies. As a result, the importance of recording, monitoring, and sharing detailed data related to suicide has been emphasized by the WHO, which will contribute to global awareness and also strengthen public health strategies [10]. However, only 38 countries have successfully implemented these measures, as of 2021 [11].

Bangladesh is a prominent LMIC in Southeast Asia, where suicide is a significant cause of unnatural deaths. However, the cases are not always reported due to various reasons, such as social stigma of mental diseases, cultural taboos, and legal implications, such as harassment by police, since attempting or committing suicide is a criminal offence in Bangladesh, and also because of cultural and religious reasons [12]. In Bangladesh, suicide gets less attention than is required as a public health concern, as the country lacks a national suicide surveillance system, which fails to provide a correct idea of the epidemiology of suicide [13].

Considering the current scenario in Bangladesh, where cases of suicide remain underreported, the availability of reliable epidemiological data on suicide is scarce in the country, which fails to bring the pressing matter to attention [14]. This lack of proper information poses a huge problem while identifying the reasons for suicide in Bangladesh. This is an area that needs urgent attention since any case of suicide not only affects a family emotionally, but also financially. Therefore, it is necessary to carry out suicide-centric research in Bangladesh to accumulate accurate data. That being the case, the Bangladesh Health and Injury Survey (BHIS) was conducted in 2003 and,d 2016 which also focused on suicide cases. Bangladesh has undergone significant social, economic, and demographic changes in the mentioned years, and thus, this study allows us to understand how suicide rates have shifted from 2003 to 2016 as a result of changes in society and public health priorities over the years. The country has experienced major transitions in its health system between 2003 and 2016, including developments in mental health policies. Therefore, comparing the data across this decade enables us to have an understanding of the impact of these changes on people’s lives. Thus, the current study compares the change in the pattern of suicides over the years in Bangladesh using data from the Bangladesh Health and Injury Survey (BHIS) of 2003 and 2016, employing a cross-sectional independent-sample design comparison.

## Methodology

### Study design and sampling

Two community-based cross-sectional health and injury surveys were carried out nationally in Bangladesh in 2003 and 2016. These surveys focused on injury mortality and morbidity. The survey that was carried out in 2016 was similar to that which was carried out in 2003. A multi-stage cluster sampling method was used in both surveys with a probability proportional to size strategy to ensure a representative sample. To ensure the sample accurately represented the nation, the data were first weighted separately for Dhaka Metropolitan City (DMC) and all other districts. Specifically, weighting within DMC accounted for slum, non-slum, and peri-urban populations, while other districts were adjusted for rural/urban populations. Finally, national estimates were calculated based on the proportional population sizes of these two main regions

For rural areas, one Upazila (sub-district) was randomly chosen from each selected district, followed by two unions (the smallest administrative unit) per Upazila, which served as sampling clusters. All households in the selected unions were included in the study. In urban areas, district headquarters and city corporations were considered, with Mohallas (neighborhood units consisting of about 400–500 households) serving as sampling clusters. A systematic sampling method was applied to select the required number of households for interviews.

In 2016, 16 districts were randomly selected. Villages were used as clusters in rural areas, with 100 villages per Upazila and 80 households per village. Urban sampling followed a systematic approach. A target sample of 70,000 households (350,000 respondents) was set, and after data cleaning and validation, information from 299,216 household residents was available for analysis.

### Data collection

Data collection was conducted using a pretested semi-structured questionnaire, primarily designed to determine the causes of injuries and deaths. The data were collected using a three-year recall period, and we compared the suicide deaths occurring in 2002 and 2015, as we found complete data for these two years from March to December.

The data were collected primarily from household heads for adult members, and information about children was collected from their mothers. If neither was available, the most knowledgeable household member was interviewed. A household member was defined as anyone living in the same house, including domestic workers and guests who stayed for an extended period.

In both surveys, verbal autopsy was used. The process of verbal autopsy involves a professional interviewer using a questionnaire to gather information from a person who knows the deceased about their signs, symptoms, and demographic traits to determine the cause of death [15].

Trained interviewers collected data on the deceased’s symptoms and demographics from a knowledgeable person. However, unlike the 2003 survey, which relied on paper-based questionnaires, the 2016 survey utilized tablets equipped with a customized data entry program, enabling real-time data upload to a server for improved data management and processing.

### Data analysis

The completeness, appropriateness, and quality of the data were examined. The Statistical Package for the Social Sciences (SPSS) software, version 27, was used to process and analyze the data. A descriptive analysis was carried out for the age and sex distribution of the population in the years 2002 and 2015. The suicidal incidence rate was calculated per 100,000 population. Suicidal incidence rate by age, sex, and place of residence was calculated with a 95% confidence interval using the number of subpopulations as denominators and the subpopulation-specific number of deaths in the specific year (2002 or 2015) as numerators. The way of committing suicide was shown as a graph.

## Results

Table 1 result shows that in 2002, the total population was 819,429, of which 402,622 were females and 416,807 were males. In 2015, the total population was 299,216, of which 149,221 were females and 149,995 were males. The individuals were also characterized according to age (2002 and 2015), the breakdown of which is provided in Table 1. Findings revealed that the majority (33% in 2002 and 35.2% in 2015) of the respondents were in the age group of 20 to 39 years.

**Table 1:** Distribution of the respondents according to age, sex, and place of residence

Variables	2002		2015	
	n	Percentage (%)	n	Percentage (%)
**Age**				
Up to 9 years	197273	24.1	51719	17.3
10–19 years	185297	22.6	61097	20.4
20–39 years	270358	33	105435	35.2
40–59 years	120344	14.7	58133	19.4
60 years and above	46157	5.6	22832	7.6
**Sex**				
Male	416,807	50.9	149,995	50.1
Female	402,622	49.1	149,221	49.9
**Place of Residence**				
Urban	384831	47	106233	35.5
Rural	434598	53	192983	64.5

**Fig. 1:**
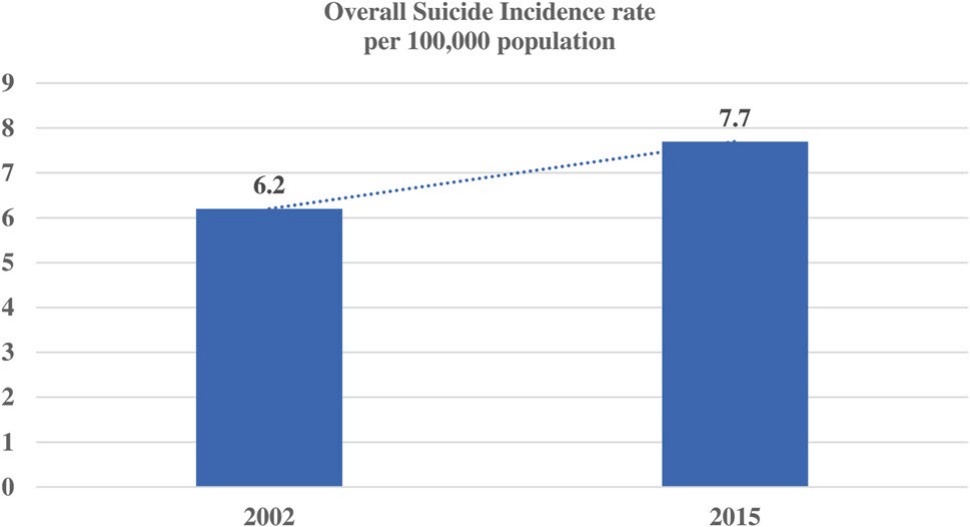
Changes in overall suicide rates over time

Calculated suicide rates showed that there is an increased rate of suicide over the decade. Analysis of these rates revealed that in 2002, the suicide rate was 6.2 per 100,000 population with 95% CI: 4.7–8.1; whereas in 2015 it increased slightly (suicide rates: 7.7, 95% CI: 5.1–11.6) (Fig. 1).

A major difference in increasing suicide rate was observed according to age in both years (2002 and 2015). The highest suicide rate was found among the 60-year-old and above age group (rate: 13, 95% CI: 2.6–23.4) in 2002. Compared to 2002, in 2015, the highest suicide rates were found among the 10 to 19-year age group (rate: 22.9, 95% CI: 13.6–38.7), though no statistically significant relationship was found. (Table 2).

**Table 2:** Suicide incidence rate by year changes according to the socio-demographic characteristics

Variables	2002		2015	
	n	Rate per 100,000 (95% CI)	n	Rate per 100,000 (95% CI)
**Age**				
10–19 years	16	8.6 (5.1–13.7)	14	22.9 (13.6–38.7)
20–39 years	24	8.8 (5.8–13)	6	5.7 (2.6–12.7)
40–59 years	5	4.2 (1.5–9.2)	1	2.8 (0.4–19.9)
60 years and above	6	13 (2.6–23.4)	2	8.8 (2.2–35.3)
**Sex**				
Male	26	6.2 (4.1–9)	7	4.7 (2.2–9.8)
Female	25	6.2 (4.1–9)	16	10.7(6.6–17.5)
**Place of Residence**				
Urban	5	1.3 (0.5–2.9)	3	2.8 (0.91–8.8)
Rural	46	10.6 (7.5–13.6)	20	10.3 (6.7–16.1)

As per sex-wise suicide rates distribution, in 2002, both males and females showed a similar rate that was 6.2 with a 95% CI: 4.1–9. But in 2015, the suicide rates among females increased almost double per 100,000 population (rate: 10.7, 95% CI: 6.6–17.5) (Table 2).

In Table 2, the result also revealed that the suicide rates were higher in rural areas compared to urban areas both in 2002 (rate: 10.6, 95% CI: 7.5–13.6) and 2015 (rate: 10.3, 95% CI: 6.7–16.1).

**Fig. 2:**
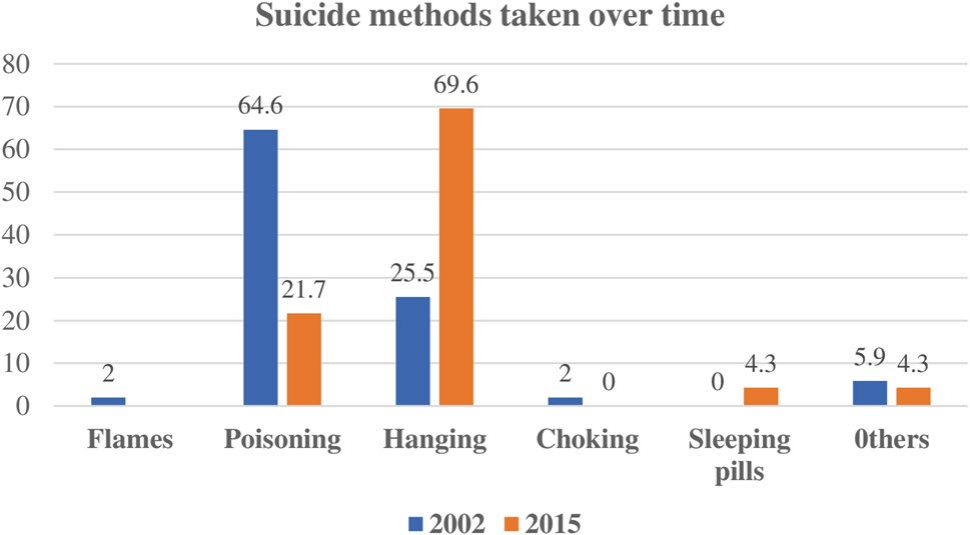
Suicide methods taken from 2002 and 2015

Over the past decade, methods of committing suicide have changed noticeably. Where poisoning was the most common (64.6%) way of suicide in 2002 and 2015, it was 21.7%. But with the change of times and years, the way of committing suicide also changes, as shown in Fig. 2. Hanging was the most common way of suicide in 2015, that is 69.6% whereas in 2002 it was only 25.5%. Another method of committing suicide in 2015 was sleeping pills, which was 4.3% (Fig. 2).

## Discussions

Bangladesh, a low socioeconomic country, experienced a concerning increase in suicide rates over a decade. Between 2002 and 2015, the rate surged from 6.2 to 7.7 deaths per 100,000 population. Studies conducted in the Southeast Asia region showed that the suicide rate was in Bangladesh in 2019, 3.85 per 100,000 population [16], in India in 2016, 16.5 per 100,000 population [17], Sri Lanka in 2022, 15 per 100,000 population [18], and Nepal in 2019, 19.67 per 100, 000 population [19] which is inconsistent with our study findings. According to WHO data, globally, the overall suicide rate was 9 per 100,000 population in 2019 [20]. Regional epidemiological data on suicide in 2019 indicated that Africa had the highest suicide rate at 11.2 per 100,000 population. This was followed by Europe at 10.5 per 100,000 population and the South-East Asia region at 10.2 per 100,000 population, respectively [16].

Numerous factors, including socioeconomic instability, low educational attainment, homelessness, divorce, drug overdose, birth rate, female labor force participation, and alcohol consumption, were linked to an increase in suicide rates over the period of time, according to epidemiological studies conducted across the world [20–24].

The increasing suicide trend in Bangladesh is primarily linked to factors such as depression or other mental health issues [12, 25], gender-based violence, and the stigma that delays women from seeking care [26, 27]. Furthermore, it is important to note that many local studies, such as those focusing on specific cohorts like rural communities [25] or university graduates [12], or those examining non-fatal outcomes like suicide attempts [27], also identify these psychiatric and socioeconomic factors as key drivers. Additionally, unemployment, low-income jobs, and poverty exacerbate this crisis [25]. Taken together, these findings align with those of other studies.

Our study findings showed that age-wise suicide rates were higher among the 60 years and above age group in 2002, that is, 13 per 100,000 population (with 95% CI: 2.6–23.4), but in 2015, the highest suicide rates were observed among adolescents (rate: 22.9 per 100, 000 population; 95% CI: 13.6–38.7). This may be because the elderly population experiences many bitter situations and different risk factors, such as loss of loved ones, physical illness, loneliness, substance use, etc., in their later life which leads them into depression, which might have a relation to suicide, which is also supported by the studies conducted globally [28, 29]. The majority of teenagers who died by suicide (90%) came from low- and middle-income nations [30]. With the change of times, Bangladesh, as well as globally, suicide rates have increased noticeably among the youth and adolescents. This study did not have the scope of exploring underlying mental health conditions of the study participants. However, some social-economic factors may precede the decision of suicidal ideation if that person already has pre-existing mental health conditions. Some studies conducted in Bangladesh found that loneliness, being bullied, depression, drug abuse, having no close friends, and social-environmental factors, such as lack of peer support and stigma of shame, may have a relation with the increase of suicide rates among the youth and young adults with pre-existing depression or other mental conditions [27, 31, 31], which is supported by the other studies conducted worldwide [32–34].

Our Data also revealed that in 2002, both sexes showed a similar suicide rate of 6.2 per 100, 000 but in 2015, the suicide rate increased approximately twice in females than in males (10.7 per 100,000;4.7 per 100,000 population). The possible reason for this result could be that women are severely affected by many socio-economic factors, like early marriage, domestic violence, gender-based violence, dowry, etc., which are the underlying cause of their severe depression and that led them to take their own lives [35]. But other research and studies conducted in different countries like Australia (17.0), Ghana (20.0), and the USA (22.4). have the highest suicidal rate in males in 2019 [16, 20, 36]. However, according to the WHO in 2016, the only countries with estimated greater rates of female suicide compared to male suicide were Bangladesh, China, Lesotho, Morocco, and Myanmar, but in 2019, suicide rates declined significantly in the above-mentioned countries [16]. In the USA, between 2001 and 2018, the overall age-adjusted suicide rate for females rose from 10.7 deaths per 100,000 standard population to a recent peak of 14.2 deaths in 2018 [37]. Studies conducted in the USA over decades demonstrated that suicide rates were continuously lowest among those aged 10–14 for both sexes and greatest among those aged 75 and older in males, 45–64 for women, during 2001–2021 [37].

According to the place of residence, in this study, participants from rural areas have a higher suicide incidence rate than those from urban areas, which is consistent with the other study findings [38–41]. In Bangladesh, suicide rates are high in rural regions due to a combination of factors. These include existing mental health conditions, younger age, lower educational attainment, low-paying jobs, a family history of mental health issues and suicide, and experiences of anxiety and insomnia [25]. Additionally, for females, notable causes include abortion and gender-based violence [26]. Hanging and poisoning were the most common ways of suicide between 2002 and 2015. Globally, hanging is one of the most common methods of committing suicide, as found in several studies. The study conducted in Australia also revealed that hanging has been the most common means of suicide over time [42]. The decrease in poisoning cases and the increase in hanging cases may be due to implementing strict regulations on the sale of pesticides in the agricultural fields, as well as poison in the market [43–45]. In certain areas of Bangladesh, such as the Jhenaidah district, suicides linked to pesticides continue to occur, and the use of class II pesticides as a means of suicide is a concerning matter [46]. The same result was found in Japan and Taiwan, where hanging is the most prevalent cause of suicide [47, 48]. But in South Korea, poisoning is the common mode of suicide among its residents [49]. Suicide depends on several factors such as the availability of resources, and this can vary based on the context and situation. In Bangladesh, obtaining any material for hanging is a lot easier and safer for anyone as long pieces of clothes are common attires in the country in the form of scarves and saree. Alternatively, obtaining poisons may be noticeable and can cause suspicion [50]. In our studies, other modes such as flames, choking, and sleeping pills are infrequently mentioned.

Though this study did not focus on the risk factors of suicide, it may serve as an important public health implication for our perspectives. Bearing in mind the increasing trend of suicide rates, makers can take different measures to overcome the situation. Bangladesh is experiencing a critical rise in suicide rates, demanding urgent, targeted national action. Interventions must focus on the most vulnerable groups—adolescents, females, and rural residents—by prioritizing research into their unique challenges. Controlling suicide methods is equally vital, including addressing the shift from poisoning to hanging and enforcing regulations on substances like Class II pesticides. Establish a strong suicide surveillance system and conduct further research to inform effective, data-driven prevention strategies.

## Conclusion

This study shows that the trends of suicide rates were increasing over time. Females, adolescents, and rural people are more prone to suicide. Over the decade, hanging was the major contributor to the suicide incidence rate. Extensive and further research is needed to explore the attributes behind all suicidal deaths.

### Strength and limitations

To determine the status and consequences of injuries, the largest community-level surveys are the Bangladesh Health and Injury Surveys of 2003 and 2016. A key strength of the study is its independence from hospital data, which typically underrepresents the true mortality rate in our nation. In Bangladesh, where the majority of the population follows the Islam religion, suicide is highly stigmatized both socially and religiously, which may lead families to under-report suicide deaths. Including relatives and neighbors in interviews somewhat reduced this issue, but some underreporting likely remains. Many individuals in developing countries, including those who attempt suicide, do not seek hospital treatment, often to avoid police involvement and legal procedures. Consequently, hospital and police records tend to underreport suicide deaths. The BHIS, one of the largest community-based injury surveys in Bangladesh, uses a substantial sample size and rigorous methodology, making its findings likely representative of national suicide statistics. The sample size differs between the two surveys because the sample size of BHIS 2003 was calculated based on the prevalence of drowning, whereas in 2016, the sample size was calculated based on the prevalence of injury. This fact resulted in a significant reduction in the sample size in BHIS 2016. This variation may have resulted in a lower capture of cases, and there is a chance of underestimating rates in the second survey. The confidence intervals for some estimates are wide, which may be due to the sample size being too small.

## Data Availability

The datasets used and/or analyzed during the current study are available from the corresponding author on reasonable request.
